# Bridged Mesoporous Oxo-Phosphonates: A General Strategy Toward Functional, Hybrid Materials

**DOI:** 10.3390/molecules30112459

**Published:** 2025-06-04

**Authors:** Elodie Gioan, Zijie Su, Yanhui Wang, Jeremy Rodriguez, Karim Bouchmella, Johan G. Alauzun

**Affiliations:** 1 ICGM, University of Montpellier, CNRS, ENSCM, 34293 Montpellier, France; elodie.gioan@umontpellier.fr (E.G.); karim.bouchmella@umontpellier.fr (K.B.); 2 Yantai Research Institute, Harbin Engineering University, 1 Qingdao Street, Development Zone, Yantai 150009, China

**Keywords:** hybrids, bisphosphonate, titania, mesoporosity

## Abstract

Combining the properties of organic and inorganic components with high surface areas and large pore volumes opens up countless possibilities for designing materials tailored to a wide range of advanced applications. As the majority of mesoporous hybrid materials are siliceous, the development of cost-effective synthetic approaches to produce water-stable hybrids with controlled porosity and functionality remains essential. Herein, we describe an original strategy for the synthesis of bridged mesoporous titania–bisphosphonate hybrids based on a one-step, template-free, non-hydrolytic sol–gel process. The reaction between Ti(OiPr)_4_ and several flexible or rigid bisphosphonate esters, in the presence of acetic anhydride (Ac_2_O) leads to the formation of TiO_2_ anatase nanorods interconnected by fully condensed bisphosphonate groups. The general method that we depict is quantitative and low cost. All materials are mesoporous with very high specific surface areas (up to 520 m^2^·g⁻^1^) and pore volumes (up to 0.93 cm^3^·g⁻^1^).

## 1. Introduction

The synthesis of porous organic–inorganic hybrid materials has been a major challenge for materials scientists for more than 30 years [1,2]. Indeed, these materials tend to combine the properties of organic and inorganic moieties with high surface areas and pore volumes. They should offer unprecedented options to design many materials adapted to a huge range of advanced applications. These hybrid materials are often separated into two different classes. In Class I hybrid materials, the organic and inorganic parts are bounded through weak bonds, whereas, in Class II hybrid materials, they are bounded toward stronger ionocovalent or covalent bonds.

Most Class II hybrid materials are siliceous derivatives, typically derived from organotrialkoxysilane coupling agents. These compounds have been extensively employed to develop a wide variety of hybrid materials exhibiting either disordered or ordered mesoporosity, including surface-functionalized silicas, (bridged) silsesquioxane gels and cogels, and periodic mesoporous organosilicas [3,4,5,6].

Research is increasingly focusing on nonsiliceous mesoporous hybrid materials to extend the applications. They are mainly developed using carboxylate (MOFs) [7,8] or phosphonate coupling agents [9,10,11,12,13].

On the past two decades, metal-organic frameworks (MOFs) have known an exponential growth [14,15]. They are notorious for their nice crystalline structures, extremely large surface areas, and well-defined micropores. However, designing mesoporous MOFs often requires elaborate strategies, and the limited water stability of MOFs might be an issue, depending on the applications [16].

On the other hand, phosphonates, known for their strong binding affinity to a broad range of metal atoms, are considered among the most promising coupling agents for designing water-stable, nonsiliceous Class II hybrid materials [17,18]. However, traditional metal phosphonates typically form dense, layered structures with inherently low porosity [19]. Various strategies have been employed to synthesize porous metal phosphonates based on titanium, zirconium, or aluminum [9,20,21,22,23]. One common approach involves the combination of bridging bisphosphonates with short monophosphonates to induce microporosity. Another method utilizes bulky multiphosphonic acids to prevent the formation of layered structures [24]. Additionally, metal phosphonates exhibiting external mesoporosity have been developed through sol–gel and/or hydrothermal techniques using bisphosphonic or multiphosphonic acids [19,25]. In these systems, the structural walls are composed of amorphous or semicrystalline metal phosphonate particles, characterized by high phosphorus-to-metal (P/M) ratios, typically ranging from about 1.3 to 2 [26,27,28]. However, the variety of functional organic groups incorporated into metal phosphonate hybrid materials remains relatively limited [29].

Therefore, establishing a template-free and versatile method for synthesizing nonsiliceous hybrids with controllable mesoporosity and customizable functionality remains a highly sought-after goal.

We have lately reported the non-hydrolytic sol–gel (NHSG) synthesis of a new family of hybrid organic-inorganic mesoporous materials with tunable porosity and functionality [30,31]. We called them “bridged mesoporous oxo-phosphonates”, or BMOPs. In this previous work, the hybrids consisted of titanium oxide nanodomains interconnected by rigid, fully condensed bisphosphonate linkers (biphenyl or bipyridine). Their porosities were shown to be easily tunable via reaction conditions, and they exhibited excellent stability across a wide pH range. We believe that the NHSG chemistry provides an elegant, simple, and powerful route to obtain hybrids with different morphologies or textures.

Herein, we report the one-pot synthesis of several hybrids containing different bisphosphonates (alkyl, aryl, and bipyridine derivatives) with TiO_2_. As we describe, this simple method, giving access to a wide family of new mesoporous hybrid materials, is generic and can be extended to different flexible or rigid bisphosphonate groups. All materials show specific surface areas between 290 and 520 m^2^ g^−1^, as well as pore volumes up to 0.93 cm^3^ g^−1^. We consider these materials to be promising as catalytic supports or sorbents given their high stability and outstanding textural properties.

## 2. Results and Discussion

In our previous work, we demonstrated that TiO_2_ with hierarchical porosity and high specific surface area can be synthesized through an NHSG reaction between Ti(OiPr)_4_ and Ac_2_O [30]. We further showed that coupling this NHSG approach with a rigid bis-diethylphosphonate precursors provides a one-step and template-free method for the synthesis of nonsiliceous hybrid materials with tunable mesoporosity and customizable post-synthetic functionalization.

**Figure 1:**
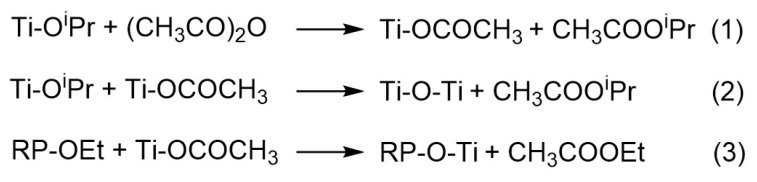
Reaction involved in the BMOP synthesis.

The synthesis of these bridged mesoporous oxo-phosphonates has been shown to be a three-step process (Equations (1)–(3) in Figure 1), where two are competitive reactions (Equations (2) and (3)). In the first step, titanium isopropoxide reacts with acetic anhydride, forming Ti-OAc intermediates (Equation (1)). These functions then react either with other titanium isopropoxide functions (Equation (2)) or with phosphonate esters (Equation (3)), where R is any functional organic group.

**Scheme 1:**
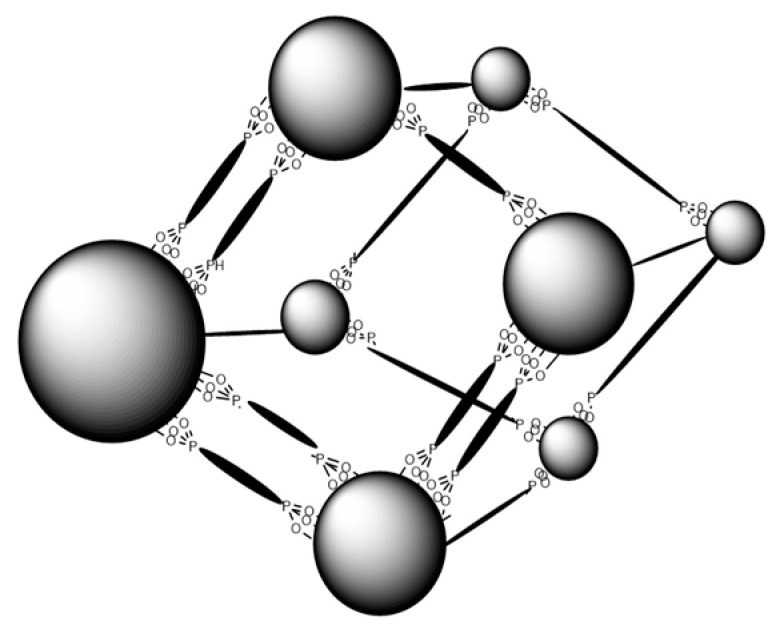
Schematic representation of BMOP (metal oxide nanoparticles bridged by bisphosphonate).

Being irreversible, TiO_2_ grows and, in time, is modified on the surface with phosphonate moieties. These simultaneous reactions form BMOP hybrids (Scheme 1).

**Figure 2:**
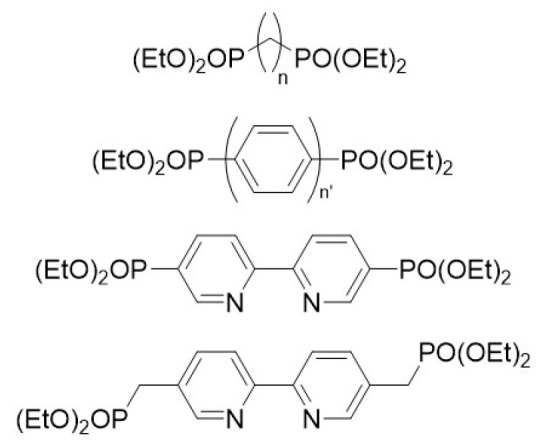
All bisphosphonates considered (n = 3, 6, and 12; n’ = 1 and 2).

A major advantage of hybrid materials is the possibility to tailor the functional organic groups to the targeted applications. In order to give access to a wide family of these new mesoporous hybrid materials with great application potential, we have considered here to keep the titania precursor and to change the organic linker while maintaining the P/Ti ratio (0.1 eq). Seven bisphosphonate precursors were studied (Figure 2). Three are flexibles: propyl (bC_3_P), hexyl (bC_6_P) and dodecyl (bC_12_P); three are rigids: phenyl (bPhP), biphenyl (bPh_2_P) and bipyridine (bPy_2_P); and one is semi-rigid: dimethylbipyrine (bPy_2_C_2_P). It is worth noting that all these molecules are commercially available but are also synthesizable.

**Scheme 2:**
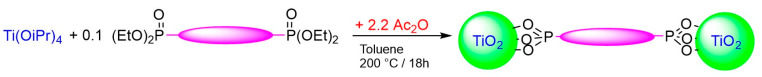
Schematic representation of BMOP syntheses.

Using a previously described procedure [30] (as depicted in Scheme 2 and developed in the experimental section), we thus obtained 7 different BMOPs: bC_3_P-Ti, bC_6_P-Ti, bC_12_P-Ti, bPhP-Ti, bPh_2_P-Ti, bPy_2_P-Ti, and bPy_2_C_2_P-Ti.

**Figure 3:**
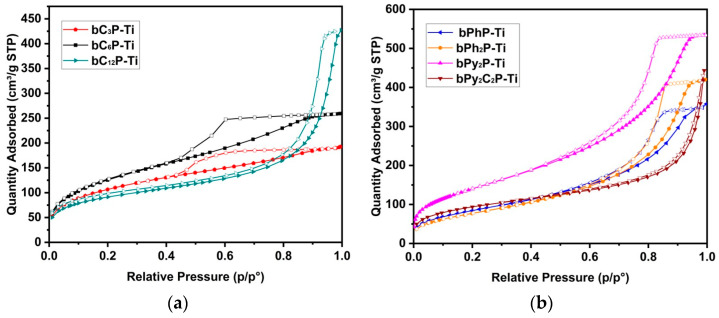
Nitrogen physisorption isotherms of flexible (**a**) and rigid (**b**) BMOP samples. Filled and open symbols refer to adsorption and desorption, respectively.

For all obtained hybrids, we measured the P/Ti ratios by MEB–EDX. According to the results, all the precursors are incorporated and the materials are homogeneous at the micrometer scale (P/Ti = 0.19 ± 0.02), within experimental error. It is worth noting that the synthetic yields are always quantitative. The nitrogen adsorption–desorption isotherms of the different samples are displayed in Figure 3a (flexible linkers) and 3b (rigid linkers and bPy_2_C_2_).

As we can observe, all samples showed significant porosity. In accordance with the IUPAC classification [32], the isotherms of theses BMOP samples are mainly of type IVa, characteristic of mesoporous adsorbents, with an H2 hysteresis loop indicating complex pore structures. The isotherms of bPy_2_C_2_ also showed Type II features (lack of plateau at high relative pressure), suggesting the presence of some macropores.

As shown in Table 1, the specific surface areas of the flexible bisphosphonates are between 330 and 470 m^2^ g^−1^. Indeed, for bC_12_P-Ti, the specific surface area is 330 m^2^ g^−1^; it is 470 m^2^ g^−1^ for bC_6_P-Ti, and it reaches 390 m^2^ g^−1^ for bC_3_P-Ti (Table 1). There is, thus, an optimal value for the bC_6_P bisphosphonate linker. A clear trend can be observed: the pore volume decreases progressively with decreasing chain length—0.30 (bC_3_P-Ti), 0.40 (bC_6_P-Ti), and 0.60 cm^3^ g^−1^ (bC_12_P-Ti). Increasing the distance between nanoparticles should indeed increase interparticle volumes.

The specific surface areas of the BMOPs containing rigid bisphosphonates are in the range of 290 m^2^ g^−1^ to 520 m^2^ g^−1^. It is somewhat more challenging to compare these data as the functions are different. Nevertheless, a decrease in specific surface area is observed from bPhP to bPh_2_P, while the pore volume shows a concomitant increase.

**Figure 4:**
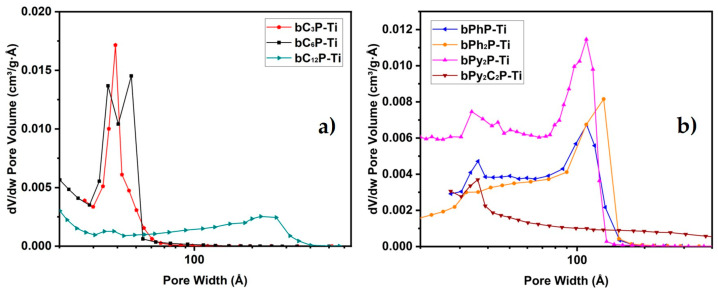
Pore sizes distribution for flexible (**a**) and rigid (**b**) BMOP samples.

The pore size distribution (Figure 4a,b) results confirmed the presence of mesopores in all BMOP samples. As depicted in these figures and summarized in Table 1, the pore diameters are centered within the range of 4.0 to 25 nm for materials with flexible linkers and around 10–13 nm for the ones with rigid linkers. The average pore diameter (estimated with BJH) is within 2.7 to 5.4 for the flexible linkers and within 6.5 to 10.9 nm for the rigid ones. As observed, the semi-rigid linker does not show evidence of porous diameters in the mesoscopic range. The porosity might be higher than 50 nm.

The peak found in the distribution at ≈4.0 nm for some samples (such as bC_6_P-Ti) is a well-known artefact. This merely indicates the presence of small pores of diameters < 4 nm, due to the instability of the meniscus at relative pressures lower than 0.42 [33].

We finally determined the Brunauer–Emmett–Teller C constant of 0.05 < P/P_0_ < 0.3, which is related to the adsorption enthalpy, for all BMOPs. The C constant represents the affinity of the solid with the adsorbate (the N_2_ molecules) and also to the heat of adsorption. The higher the value of C, the higher the interaction [32,34]. The highest C values were recorded for the flexible linkers, although all values remain within the 46–134 range.

Relatively similar C constant values (≈30) were previously reported for nanoparticles grafted by octylphosphonic acid [35].

TiO_2_ prepared by the exact same non-hydrolytic procedure in toluene, is purely mesoporous, with a pore volume of 0.36 cm^3^ g^−1^ and a specific surface area of 170 m^2^ g^−1^ [36]. As previously described, the presence of bridging bisphosphonate groups leads to the formation of a high specific surface area. Thus, this general method allows one to obtain highly mesoporous material with all studied bisphosphonates.

**Figure 5:**
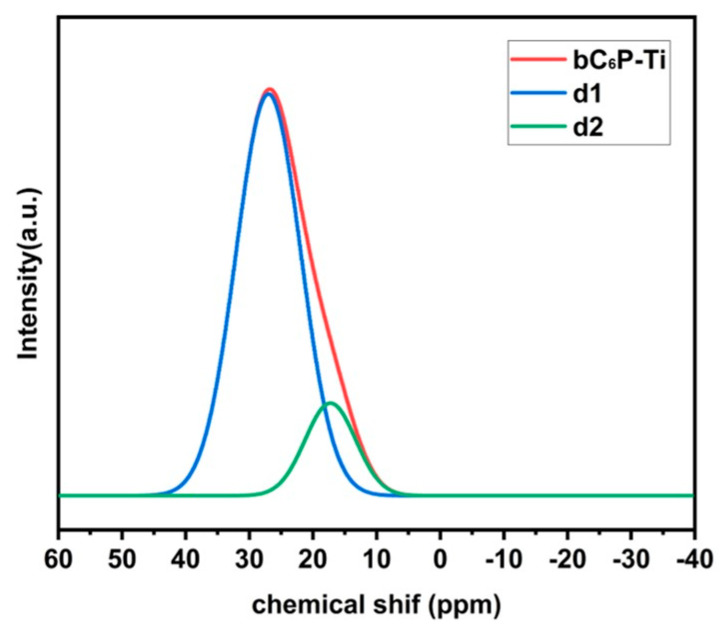
^31^P CP magic angle spinning solid-state NMR spectrum of the bC_6_P-Ti hybrid materials. The d1 and d2 curves represent the deconvolution of the bC_6_P-Ti curve.

The ^13^C MAS NMR (Figure S1) performed on bC_12_P-Ti (a) and bPy_2_P-Ti (b) samples confirmed the incorporation of the organic linkers without alteration during the synthetic process. As observed in the spectra, the absence of resonances at ≈60 ppm (OCH_2_CH_3_ sites) and ≈75 pmm (OCH(CH_3_)_2_ sites) reveals the full condensation of P-OEt and Ti(OiPr) groups. We can notice the presence of two signals centered at 23 ppm and 178 ppm, pointing to the presence of residual acetate groups within the materials. ^31^P MAS solid-state NMR spectrum (Figure 5) of the bC_6_P-Ti hybrid material displays a very broad signal in the 10 to 40 ppm range, centered at 27 ppm.

We have reported similar broad resonances for TiO_2_–octylphosphonate hybrid materials prepared in the same one-step NHSG process, starting from Ti(OiPr)_4_ and H_3_C-(CH_2_)_7_-PhPO(OEt)_2_ [35]. These peaks confirm the presence of phosphonate species bounded to the TiO_2_ particles through Ti–O–P bonds. The lack of a sharp resonance around 7 ppm demonstrates the absence of a layered titanium bisphosphonate phase. The deconvolution determined by OriginPro software (2022b SR1; v9.9.5.171) shows two contributions at 27 ppm (85%) and 17.3 ppm (15%). As previously demonstrated. this is consistent with the tridentate bonding of R-PO_3_ onto the titania surfaces [35].

Electron microscopy images (Figure S2) show that the bPh_2_P-Ti material appeared to be formed of densely aggregated, roughly spherical particles.

**Figure 6:**
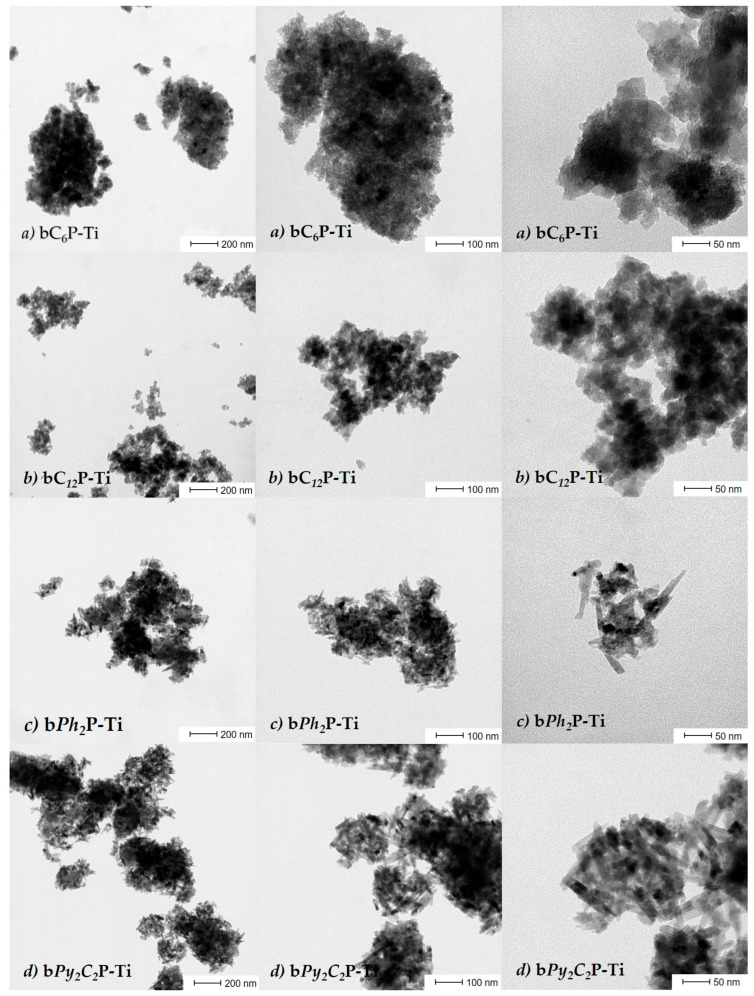
TEM images with scale bars at 50, 100 and 200 nm; of bC_6_P-Ti (**a**), bC_12_P-Ti (**b**), bPh_2_P-Ti (**c**), and bPy_2_C_2_P-Ti (**d**) samples.

As shown in the TEM images (Figure 6), at lower scale, the particles form a three-dimensional mesoporous network, demonstrating the generality of our synthetic route. Interestingly, BMOPs obtained from a rigid linker appears to be constituted of TiO_2_ nanorods (Figure 6c,d), while the ones prepared from flexibles linker (bC_6_P-Ti and bC_12_P-Ti) exhibit an assembly of spherical nanoparticles (10 ± 5 nm diameter). The nanorods have a length of 60 ± 15 nm and 10 ± 2 nm width. The morphology of the samples is thus significantly dependent on the organic linker. At this nanometer scale, the sample textures are thus significantly dependent on the rigidity of the organic linker. We do not have at this time an explanation for these interesting differences.

**Figure 7:**
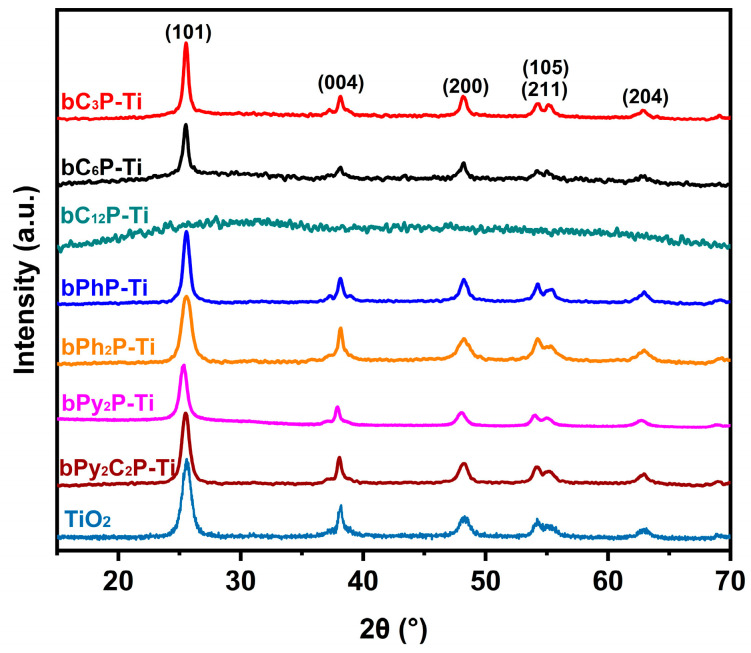
Powder X-ray diffraction (XRD) patterns of BMOP and TiO_2_ samples.

The X-ray diffraction (XRD) patterns of the hybrid samples and TiO_2_ are presented in Figure 7.

The samples’ crystallinities do not differ much, except for the bC_12_P-Ti sample, which exhibits amorphous structure. The patterns of the BMOP hybrids showed the presence of anatase nanocrystals (JCPDS 21-1272). There was no evidence of any rutile phase. In all cases and as previously described, low-angle XRD showed no evidence for layered titanium bisphosphonate phase. Given that this stable phase is generally non-porous, its formation could adversely affects organic group accessibility and compromises the material’s textural characteristics. The sizes of the anatase crystallites determined by the Debye–Scherrer equation from the (101) and (200) peaks of the powder X-ray diffraction patterns vary from 8.5 to 14.6 nm. As observed, TiO_2_ nanomaterials synthesized with the exact same procedure have the same anatase phase.

The attenuated total reflection (ATR)—Fourier transform infrared (FTIR) spectra of hybrid materials display a single broad band between 1000 and 1100 cm^−1^, attributed to the vibrational modes of R-PO_3_ tetrahedra (Figure S3). The absence of characteristic bands at ≈1220 cm^−1^ (P=O stretching vibration) and ≈950 cm^−1^ (P–OC stretching vibrations) [37] indicates that the phosphonate groups adopt a tridentate coordination environment, similar to that found in layered titanium phosphonates. As previously observed in sol–gel-derived TiO_2_–phenylphosphonate hybrid materials, P atoms are bonded to three Ti atoms in CP(OTi)_3_ sites [38]. Vibrations observed in the 1400–1500 cm^−1^ region are assigned to CH_3_ and CH_2_ deformations associated with residual organic groups on the surface (e.g., Ti–OiPr, Ti–O–CMePhOiPr), as well as CH_3_, CH_2_, and P–CH_2_ deformations originating from the alkylphosphonate linkers (C_3_, C_6_, and C_12_). The three bands between 2850 and 3000 cm^−1^ are ascribed to the C–H sp3 symmetric and asymmetric stretching vibrations of bonds in CH_2_ and CH_3_, mostly in the dodecyl unit. The intensity of these bands is directly related to the amount of Csp_3_.

A weak, broad band between 3000 and 3800 cm^−1^, characteristic of O–H stretching vibrations, indicates the presence of a small amount of adsorbed water, corroborated by the band at 1620 cm^−1^, corresponding to its bending mode. This broad feature may also originate from hydroxyl groups generated by the hydrolysis of residual surface species during the washing process or manipulation under air.

In order to confirm the incorporation of bisphosphonate units and to determine the degree of condensation, we undertook thermogravimetric measurements of all BMOP materials from room temperature up to 650 °C. We rescaled all data curves to 0% mass lost at 150 °C. According to TGA (Figure S4 for flexible BMOPs and Figure S9 for rigid BMOPs), all BMOPs gradually lose mass upon increasing the temperature up to 650 °C. This loss is mainly due to the decomposition related to carbon and hydrogen oxidation, as well as the oxidation of nitrogen, resulting in the release of nitrogen oxides (NO_x_). The major decomposition range is within the range of 300–500 °C. This demonstrates that these materials do not present higher stability under air than siliceous hybrids materials.

The lost mass is in good agreement with the total number of organic units inserted into the hybrids. The values are between 12 and 31% for bC_3_P-Ti and bC_12_P-Ti. The crosslinking rate or degree of condensation lies between 82 and 96%. This result is consistent with the ^13^C data (no remaining ethoxy groups and few Ti-Ac).

In order to study the stability of these hybrids, we immersed the bC_12_P-Ti material for 24 h at room temperature at pH 3 and at pH 11 under stirring. After further rinsing and drying steps (same conditions as before), the materials were characterized by nitrogen sorption. The isotherms are presented in the “additional information” section (Figure S5). As can be seen, there is no change in the mesoporosity part of the isotherm (type IV, H2). There is, however, a reduction in the microporosity section and, therefore, a significant decrease in specific surface area (approx. 50%). Indeed, the surface area drops from 330 m^2^ g^−1^ to 168 m^2^ g^−1^ (pH 3) and 162 m^2^ g^−1^ (pH 11). Pore volumes are reduced by 20% (from 0.66 cm^3^ g^−1^ to 0.51 cm^3^ g^−1^ and 0.50 cm^3^ g^−1^_,_ respectively). Hybrids obtained with flexible precursors therefore appear to be slightly less stable than those obtained with rigid precursors [30].

## 3. Materials and Methods

Titanium (IV) isopropoxide (Ti(OiPr)_4_, 97%) and acetic anhydride (Ac_2_O, 99%) were purchased from Sigma-Aldrich (St. Quentin Fallavier, France). Tetraethyl 1,3 propyl-bisphosphonate (bC_3_P, 95%), tetraethyl 1,6 hexyl-bisphosphonate (bC_6_P, 95%), tetraethyl 1,12 dodecyl-bisphosphonate (bC_12_P, 95%), tetraethyl 1,4 phenyl-bisphosphonate (bPhP, 97%), tetraethyl 1,1′-biphenyl-4,4′-bisphosphonate (bPh_2_P, 97%), tetraethyl 2,2′-bipyridine-5,5′-bisphosphonate (bPy_2_P, 97%), and tetraethyl 2,2′-bipyridine-5,5′-dimethylbisphosphonate (bPy_2_C_2_P, 95%) were purchased from SiKEMIA (Montpellier, France).

All reactants were used without further purification. Toluene (Sigma-Aldrich 99.7%) was obtained over a Pure Solve MD5 solvent purification system (H_2_O < 10 ppm) and controlled with a Karl Fischer coulometer.

### 3.1. Synthesis of Hybrid Materials

To avoid the presence of water, the addition of reactants and the sealing of the autoclaves were carried out in a glovebox under an argon atmosphere (<10 ppm of H_2_O and O_2_).

In a typical synthesis, titanium (IV) isopropoxide (1.50 g, 5.25 mmol) was solubilized in toluene (8 mL), before adding the bisphosphonate (0.53 mmol). After 10 min of stirring, the acetic anhydride (2.2 eq. 1.18 g, 11.55 mmol) was added slowly. After stirring for another 10 min, the obtained solution was transferred to a stainless-steel autoclave (Parr Instruments) with a PTFE lining (23 mL), which was then sealed. The autoclave (fill rate of approx. 45%) was heated in an oven at 200 °C under autogenous pressure for 18 h. After reaction, the resulting monolithic gel was thoroughly washed with hot ethanol (Soxhlet for 24 h) and acetone (5 × 30 mL). The gel was dried under reduced pressure (100 Pa) at 120 °C and finally ground into a fine powder for characterization.

### 3.2. Characterization Methods

Fourier transform infrared (FTIR) spectra were recorded in attenuated total reflection (ATR) mode using a Spectrum II spectrometer (PerkinElmer, ICGM, Montpellier, France).

Powder X-ray diffraction (XRD) patterns were recorded using a Bruker D8 diffractometer with CuKα radiation (λ = 0.15418 nm), using a Ni filter to suppress CuKβ radiation.

Scanning electron microscopy (SEM) images were acquired on a Hitachi S-4800 microscope and energy-dispersive X-ray spectroscopy (SEM–EDX) was conducted using an Oxford Instruments X-MaxN SDD detector (ICGM, Montpellier, France).

Solid-state ^31^P magic angle spinning (MAS) NMR experiments were performed on a Varian VNMRS 400 MHz (9.4 T) spectrometer equipped with a 3.2 mm T3 HXY MAS probe. Single-pulse experiments were carried out with a spinning speed of 20 kHz, a 90° excitation pulse of 3 μs, a recycle delay of 30 s, and SPINAL-64 ^1^H decoupling at 100 kHz. Two hundred transients were accumulated. The ^31^P chemical shifts were referenced externally to hydroxyapatite (Ca_10_(PO_4_)_6_(OH)_2_) at 2.8 ppm relative to 85% H_3_PO_4_.

Solid-state ^13^C cross-polarization magic angle spinning (CPMAS) NMR spectra were recorded on a Varian VNMRS 300 MHz spectrometer using a 3.2 mm double-resonance T3 probe, with a spinning speed of 12 kHz.

The textural properties of the materials were investigated by nitrogen adsorption–desorption measurements at −196 °C using a 3Flex surface analyzer (Micromeritics). Prior to analysis, the samples were degassed under vacuum (1.33 × 10^−3^ mbar) at 250 °C for 15 h. Specific surface areas (SSAs) were determined using the Brunauer–Emmett–Teller (BET) model within a relative pressure (P/P_0_) range of 10^−5^ to 0.1. The total pore volume was estimated from the amount of nitrogen adsorbed at a relative pressure of 0.99. Microporous surface area and volume (for pores with widths w < 2 nm) were evaluated using the t-Plot method in the linear P/P_0_ range of 0.2–0.5. Pore size distributions were determined by the Barrett–Joyner–Halenda (BJH) method, applied to the desorption branch for pores in the 2–50 nm range, and by the Density Functional Theory (DFT) method for pores in the 1–5 nm range.

The thermal stability of the materials was assessed by thermogravimetric analysis (TGA) under synthetic air using a STA 409 PC Luxx thermal analyzer (Netzsch, ICGM, Montpellier, France). Samples were placed in alumina crucibles and heated to 650 °C at a rate of 5 °C·min^−1^.

## 4. Conclusions

In summary, we describe a simple, general, low-cost, and original synthesis of hybrid mesoporous materials via a template-free non-hydrolytic route. These hybrids contain TiO_2_ nanodomains bridged by different bisphosphonates. All materials obtained have high specific surface areas and large pore diameters, with either flexible or rigid linkers. The unique combination of accessible functionality and outstanding aqueous stability makes these bridged mesoporous titania–bisphosphonates promising materials. This family of hybrids is complementary to existing organosilicas, MOFs, and metal phosphonates, depending on uses. This opens exciting opportunities in various advanced applications, such as heterogeneous catalysis, drug delivery, ion exchange and adsorption/separation. Their effectiveness in these applications will be investigated in due course.

## Figures and Tables

**Table 1:** Recapitulative data from N_2_ sorption, XRD, and TGA measurements.

Sample	S_BET_^a^ (m^2^ g^−1^)	V_p_^b^ (cm^3^ g^−1^)	V_m_^c^ (cm^3^ g^−1^)	D_p_^d^ (nm) BJH	D_p_^e^ (nm)	C_BET_^f^	d_cryst_^g^ (nm)	Crosslinking Rate (%)
bC_3_P-Ti	390	0.30	<0.01	4.0	4.0	72	14.6	96
bC_6_P-Ti	470	0.40	<0.01	2.7	4.0	56	11.7	89
bC_12_P-Ti	330	0.66	0.09	5.4	25	134	amorphous	89
bPhP-Ti	315	0.54	<0.01	9.6	13	54	12.8	96
bPh_2_P-Ti	290	0.65	<0.01	7.1	13	47	8.5	96
bPy_2_P-Ti	520	0.93	0.06	6.5	10	46	8.9	86
bPy_2_C_2_P-Ti	345	0.69	0.05	10.9	-	79	10.1	97

^a^ BET specific area. ^b^ Total pore volume at P/P_0_ = 0.99. ^c^ Volume of micropores estimated by DFT analysis. ^d^ BJH average pore diameter in the 2 to 50 nm range from the desorption branch. ^e^ Pore diameter in the 2 to 50 nm range of the desorption branch. ^f^ Brunauer–Emmett–Teller C constant of 0.05 < P/P_0_ < 0.3. ^g^ Crystallite size estimated by the Scherrer equation.

## Data Availability

The data presented in this study are available on request from the corresponding author.

## Supplementary Materials

The following supporting information can be downloaded at: https://www.mdpi.com/article/10.3390/molecules30112459/s1, Figure S1: ^13^C MAS NMR magic angle spinning solid-state NMR spectra of the bC_12_P-Ti and bPy_2_P-Ti hybrid materials; Figure S2: SEM images of bPh_2_P-Ti; Figure S3: ATR-FTIR spectra of the BMOP hybrid materials; Figure S4: TGA curves of flexible (a) and rigid (b) BMOPs.
