# Supplementary material for: Bridged Mesoporous Oxo-Phosphonates: A General Strategy Toward Functional, Hybrid Materials
Source: Molecules. 2025 Jun 4;30(11):2459. doi: 10.3390/molecules30112459 (PMC12157266; doi:10.3390/molecules30112459)
Supplement: Supplementary file 1 [file molecules-30-02459-s001.zip › molecules-3647749-supplementary.pdf]

molecules

MDPI

Supplementary information

Bridged Mesoporous Oxo-Phosphonates: A General Strategy Toward Functional, Hybrid Materials

Elodie Gioan[1], Zijie Su[1], Yanhui Wang[2], Jeremy Rodriguez[1], Karim Bouchmella[1] and Johan G. Alauzun[1*]

[1] ICGM, University of Montpellier, CNRS, ENSCM, 34293 Montpellier, France
[2] Yantai research institute, Harbin engineering university, 1 Qingdao Street, Development Zone, Fushan District, Yantai City, Shandong Province, China
* Correspondence: johan.alauzun@umontpellier.fr

Academic Editor: Firstname Last-name

Received: date
Revised: date
Accepted: date
Published: date

Citation: To be added by editorial staff during production.

Copyright: © 2025 by the authors. Submitted for possible open access publication under the terms and conditions of the Creative Commons Attribution (CC BY) license (https://creativecommons.org/licenses/by/4.0/).

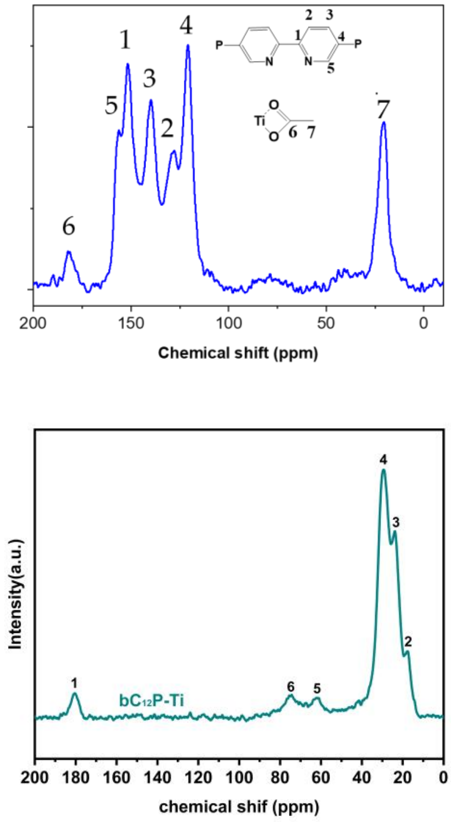

Figure S1. ^{13}C MAS NMR magic angle spinning solid-state NMR spectra of the $bC_{12}P$-Ti and bPy_2P-Ti hybrid materials

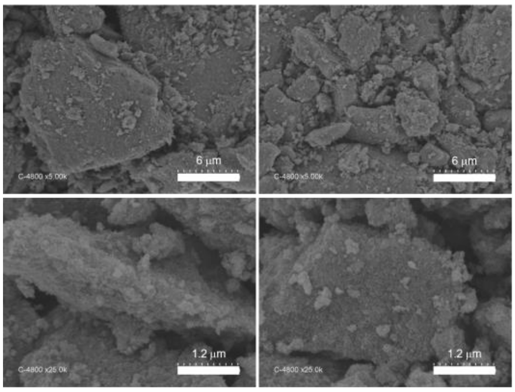

Figure S2. SEM images of bPh_2P-Ti

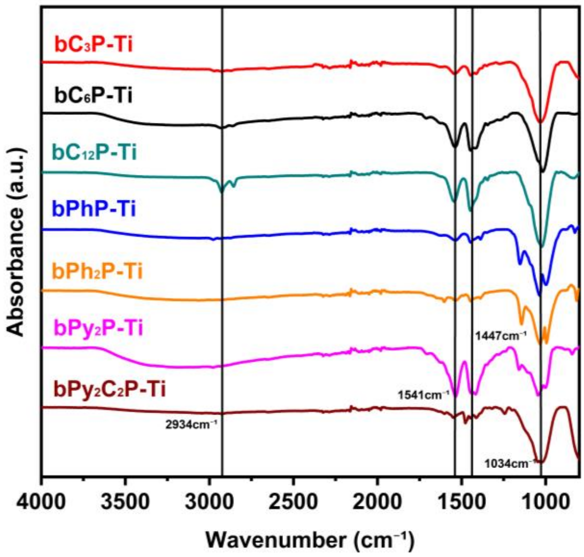

Figure S3. ATR-FTIR spectra of the BMOP hybrid materials.

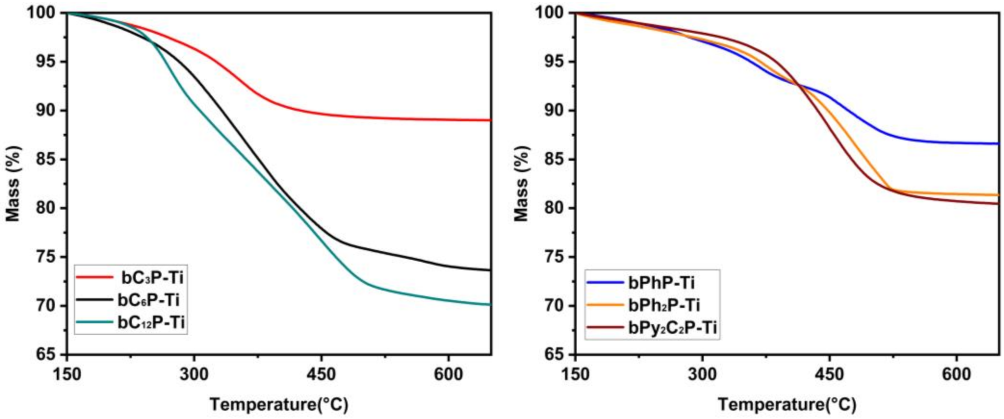

Figure S4. TGA curves of flexible (a) and rigid (b) BMOP.

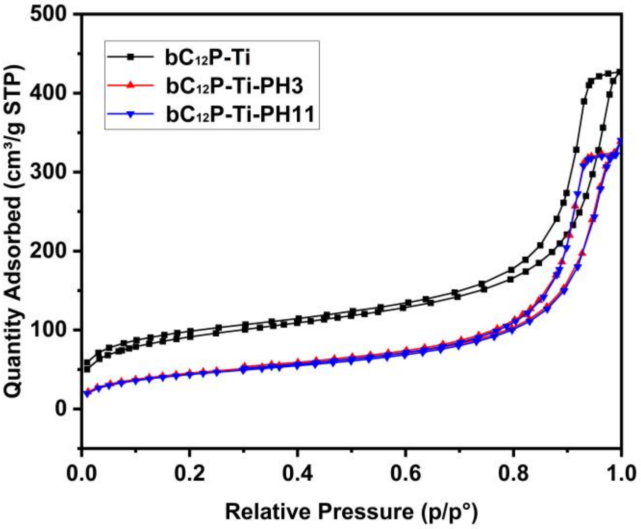

Figure S5. Nitrogen physisorption isotherms of $bC_{12}P$-Ti samples. Filled and open symbols refer to adsorption and desorption, respectively.
